# Predictors and estimation of risk for early exit from working life by poor health among middle and older aged workers in Korea

**DOI:** 10.1038/s41598-018-23523-y

**Published:** 2018-03-26

**Authors:** Wanhyung Lee, Jin-Ha Yoon, Jung-Wan Koo, Sei-Jin Chang, Jaehoon Roh, Jong-Uk Won

**Affiliations:** 1 The Institute for Occupational Health, College of Medicine, Yonsei University, Seoul, Korea; 2 Graduate School of Public Health, College of Medicine, Yonsei University, Seoul, Korea; 3 Incheon Worker’s Health Center, Incheon, Korea; 4 Department of Preventive Medicine, College of Medicine, Yonsei University, Seoul, Korea; 5 Department of Occupational and Environmental Medicine, Seoul St. Mary’s Hospital, College of Medicine, The Catholic University of Korea, Seoul, Korea; 6 Department of Preventive Medicine, Institute Occupational and Environmental Medicine, Yonsei University Wonju College of Medicine, Wonju, Korea

## Abstract

The aims of this study were to investigate the predictors and estimate the risk for early exit from work owing to poor personal health status of the retirees. This study analysed the longitudinal data of 2,708 workers aged more than 45 years old from the Korean Longitudinal Study of Ageing. Multivariate Cox regression analyses were conducted to identify the predictors and to build a prediction model for early exit from work due to poor health. Internal validation was performed using random split, and external validation using the English Longitudinal Study of Ageing. Over the 8-year follow-up, 124 workers exited work early because of poor health. Significant predictors for early exit from work due to poor health included hypertension (hazard ratio [HR], 1.52; 95% confidence interval [CI], 1.01–2.28), abnormal body mass index (HR, 1.60; 95% CI, 1.10–2.35), decreased grasping power index, and perceived health status. The prediction model designed to estimate the risk of unwanted early exit from work because of poor health status showed fair performance in both the internal and external validations. The current study revealed the specific determinants and the possibility of prediction of shortened working life due to poor health status.

## Introduction

Ageing workers have increased both in number and in proportion among the working population; this has led to significant economic and public health challenges worldwide. The main reasons for this increase in ageing workers include the increasing life expectancy and decreasing birth rates^1^. The International Labour Organization has estimated that, by 2025, the proportions of the working population aged ≥55 years will be 21%, 32%, 30% and 17% in Asia, Europe, North America, and Latin America, respectively^2^.

The Republic of Korea is one of most rapidly aging countries in the world. The Korean population’s average life expectancy has increased from 72 years in 1990 to 84 years in 2014. As seen globally, the increasing age among Koreans has resulted in an increasing elderly working population. The era of ‘homo-hundred’ is almost upon us. However, an increasing older population without social or economic activity such as a job is directly associated with heavy economic and public health burdens^3^. Thus, several countries have established policies focused on lengthening the working life, and the determinants of early exit from work among aging workers are hence of interest^4^.

Early exit from work without sufficient financial preparation could bring a financial crisis to both the workers and their family^5^. Especially, workers who cannot continue their working life due to decreased work ability secondary to personal health problems are an important concern in the field of occupational health^6^. Thus, it is important to understand why workers exit work early, especially in terms of detectable and preventable public health risk factors, in an ageing society. To educate workers regarding the possibility of unwanted early exit from work due to poor health is a fundamental strategy for maintaining the working population in an ageing society.

However, until now, very little attention has been paid to health conditions linked to shortening the working life among workers. Instead, most studies in the field of early exit from work have focused on the roles of pension and economic status in making this decision. Especially, in Korea, many workers retire early because of poor health conditions^7^. Fortunately, pioneering existing research has recognized the critical role of the workers’ health status on a wide range of processes related to early exit from workplace, using representative data of the Korean elderly population^8^.

According to the above-mentioned study, both chronic disorders (hypertension and diabetes) and unhealthy behaviours (smoking and obesity) are principal determining factors of unwanted early exit from work due to health problems. Thus, the present research attempted to analyse the impact of the determinants reported in the previous study (chronic disorders and unhealthy behaviours), perceived health-related factors, and working conditions on early exit from work among Korean workers. We also assessed the probabilities of early exit from work due to the workers’ health condition, which may be helpful to ensure a longer working life among older workers. Finally, we established a prediction model of early exit from work due to the workers’ personal health status.

## Results

The basic characteristics of the study participants at baseline are presented in Table 1. There were 2,708 participants in our sample, including 124 (4.6%) participants with early exit from work due to poor health (59 men and 65 women), over the 8 years of follow-up. The mean (standard error) ages at baseline were 56.54 (±0.53) and 52.34 (±0.11) years for participants who did and did not exit work early due to poor health, respectively.

**Table 1:** Characteristics of the study participants at baseline.

	Early exit from work due to poor health [n (%) or mean (±standard error)]		*p* value
	No	Yes	
No. of participants	2,584 (95.42)	124 (4.58)	
Age (years)	52.34 (±0.11)	56.54 (±0.53)	<0.0001
Sex			<0.0001
Men	1,779 (96.79)	59 (3.21)	
Women	805 (92.53)	65 (7.47)	
Marital status			0.1905
Married	2,324 (95.60)	107 (4.40)	
Divorced, separated, or never	260 (93.86)	17 (6.14)	
Education			<0.0001
Middle school	994 (93.16)	73 (6.84)	
High school	1,079 (96.77)	36 (3.23)	
College or university	511 (97.15)	15 (2.85)	
Household income ($)			0.0007
<20,000	992 (93.67)	67 (6.33)	
<30,000	498 (95.95)	21 (4.05)	
<40,000	479 (96.57)	17 (3.43)	
≥40,000	615 (97.00)	19 (3.00)	
Type of work			0.1409
Paid worker	1,452 (94.90)	78 (5.10)	
Self-employed	1,132 (96.10)	46 (3.90)	
Occupational classification			0.1849
Higher-skilled white collar	291 (96.04)	12 (3.96)	
Lower-skilled white collar	413 (96.95)	13 (3.05)	
Pink collar	595 (94.59)	34 (5.41)	
Green collar	173 (95.58)	8 (4.42)	
Skilled blue-collar	606 (96.04)	25 (3.96)	
Unskilled blue-collar	506 (94.05)	32 (5.95)	
Size of enterprise			0.1863
<30 or one-man company	1,728 (95.05)	90 (4.95)	
>30 or hired employee	856 (96.18)	34 (3.82)	
Time of work			0.1688
Part-time	240 (97.17)	7 (2.83)	
Full-time	2,344 (95.25)	117 (4.75)	
Smoking			0.0221
Never or past	1,747 (94.79)	96 (5.21)	
Current	837 (96.76)	28 (3.24)	
Alcohol consumption			0.9530
Never or social	2,183 (95.41)	105 (4.59)	
Heavy	401 (95.48)	19 (4.52)	
Exercise status			0.0425
None	1,535 (94.75)	85 (5.25)	
Regular	1,049 (96.42)	39 (3.58)	
Hypertension			<0.0001
No	2,215 (96.26)	86 (3.74)	
Yes	369 (90.66)	240 (9.34)	
Diabetes			0.0087
No	2,359 (95.72)	107 (4.28)	
Yes	189 (91.75)	17 (8.25)	
Obesity or underweight			0.0034
No	1,912 (96.13)	77 (3.87)	
Yes	672 (93.46)	47 (6.54)	
Depression			0.0447
No	2,158 (95.78)	95 (4.22)	
Yes	426 (93.63)	29 (6.37)	
Grasping power index	31.66 (±0.16)	26.55 (±0.66)	<0.0001
Perception factor scores			
Health status	6.68 (±0.04)	5.67 (±0.22)	<0.0001
Economic status	5.42 (±0.04)	4.96 (±0.21)	0.0319
Quality of life	6.68 (±0.04)	6.44 (±0.15)	0.1174
Working life expectancy	8.10 (±0.05)	6.83 (±0.29)	<0.0001

In terms of socioeconomic status, the highest proportions of early exit from work due to poor health were reported in those with an education status of less than middle school graduation (6.84%) and among participants with the lowest household income level (6.33%), with statistical significance (*p* < 0.0001 and *p* = 0.0007, respectively).

Regarding the occupational characteristics, regular paid workers showed a higher proportion of early exit from work due to poor health than those who were self-employed. Especially, unskilled blue-collar workers had the highest prevalence of early exit from work due to poor health (5.95%), but there was no statistical significance. Moreover, non-significant tendencies of higher prevalence of early exit from work due to poor health were noted in small-size enterprises (<30 workers and one-man companies) and full-time workers.

In terms of health status, participants who did not exercise regularly showed a higher prevalence of early exit from work due to poor health than their counterparts. Furthermore, participants with hypertension, diabetes, abnormal BMI, and lower grasping power index demonstrated significantly higher prevalence rates of unwanted early exit from work.

For the perception status, the participants with early exit from work due to poor health showed significant lower mean scores in the health, economic, and working life expectancy statuses.

Table 2 shows the risks for early exit from work due to poor health according to several covariates. Significantly decreased risks of early exit from work due to poor health were found to correlate with increased education (HR, 0.49–0.44) and household income levels (HR, 0.65, 0.55, and 0.49, respectively) in the univariate analyses.

**Table 2:** Results from the univariate and multivariate Cox regression analyses of early exit from work due to poor health.

	Cases (n)	Person-years	Early exit from work due to poor health, Hazard Ratio (95% CI)	
			Crude	Fully adjusted
Age (years)	—	—	**1**.**14** (**1**.**11–1**.**18**)	**1**.**15** (**1**.**10–1**.**19**)
Sex				
Men	59	295	reference	reference
Women	65	244	**2**.**49** (**1**.**75–3**.**54**)	1.63 (0.81–3.26)
Marital status				
Married	107	467	reference	reference
Divorced, separated, or never	17	72	1.58 (0.95–2.64)	1.23 (0.71–2.12)
Education (graduation)				
Middle school	73	311	reference	reference
High school	36	158	**0**.**49** (**0**.**31–0**.**68**)	1.11 (0.70–1.77)
College or university	15	70	**0**.**44** (**0**.**25–0**.**78**)	1.10 (0.53–2.33)
*p* value for linear trend			0.0001	0.7157
Household income ($)				
<20,000	67	298	reference	reference
<30,000	21	74	**0**.**65** (**0**.**40–1**.**06**)	1.01 (0.61–1.70)
<40,000	17	81	**0**.**55** (**0**.**33–0**.**94**)	0.99 (0.56–1.75)
≥40,000	19	86	**0**.**49** (**0**.**29–0**.**81**)	0.78 (0.43–1.44)
*p* value for linear trend			0.0016	0.5242
Type of work				
Paid worker	78	332	reference	reference
Self-employed	46	207	**0**.**59** (**0**.**38–0**.**90**)	0.70 (0.49–1.01)
Occupational classification				
Higher-skilled white collar	12	60	reference	reference
Lower-skilled white collar	13	47	0.70 (0.32–1.54)	0.41 (0.17–1.00)
Pink collar	34	135	1.22 (0.63–2.36)	0.55 (0.24–1.30)
Green collar	8	39	0.86 (0.35–2.10)	**0**.**28** (**0**.**09–0**.**83**)
Skilled blue-collar	25	119	0.90 (0.45–1.79)	0.57 (0.25–1.33)
Unskilled blue-collar	32	139	1.39 (0.72–2.71)	**0**.**42** (**0**.**18–0**.**99**)
Size of enterprise				
≤30 or one-man company	90	390	reference	reference
>30 or hired employer	34	149	0.82 (0.55–1.22)	1.06 (0.69–1.64)
Time of work				
Part-time	7	23	reference	reference
Full-time	117	516	**2**.**59** (**1**.**17–5**.**72**)	1.47 (0.69–3.15)
Smoking				
Never or past	96	404	reference	reference
Current	28	135	**0**.**61** (**0**.**40–0**.**94**)	1.08 (0.66–1.79)
Alcohol consumption				
Never or social	105	468	reference	reference
Heavy	19	71	0.98 (0.60–1.59)	1.18 (0.69–2.00)
Exercise status				
None	39	180	reference	reference
Regular	85	359	**0**.**67** (**0**.**46–0**.**98**)	0.70 (0.47–1.06)
Hypertension				
No	86	381	reference	reference
Yes	38	158	**2**.**60** (**1**.**78–3**.**82**)	**1**.**52** (**1**.**01–2**.**28**)
Diabetes				
No	107	454	reference	reference
Yes	17	85	**1**.**87** (**1**.**12–3**.**12**)	1.31 (0.77–2.23)
Obesity or underweight				
No	77	330	reference	reference
Yes	47	209	**1**.**69** (**1**.**18–2**.**43**)	**1**.**60** (**1**.**10–2**.**35**)
Depression				
No	95	428	reference	reference
Yes	29	111	1.51 (1.00–2.29)	0.94 (0.60–1.49)
Grasping power index	—	—	**0**.**93** (**0**.**91–0**.**95**)	**0**.**95** (**0**.**91–0**.**98**)
Perception factor scores				
Health status	—	—	**0**.**81** (**0**.**75–0**.**87**)	**0**.**86** (**0**.**78–0**.**95**)
Economic status	—	—	**0**.**92** (**0**.**85–0**.**99**)	0.98 (0.88–1.09)
Quality of life	—	—	0.93 (0.84–1.02)	1.09 (0.96–1.24)
Working life expectancy	—	—	**0**.**85** (**0**.**80–0**.**89**)	0.94 (0.88–1.00)

Bold values indicate statistical significance.

The associations of older age, hypertension, and abnormal BMI with increased risk for early exit from work due to poor health remained in the multivariate analysis, with HRs (95% CIs) of 1.15 (1.10–1.19), 1.52 (1.01–2.28), and 1.60 (1.10–2.35), respectively. Moreover, increased grasping power index and perceived health status score were significantly associated with reduced risks of early exit from work due to poor health.

On the other hand, no significant association was observed between early exit from work due to poor health and marital status, enterprise size, alcohol consumption, or perceived quality of life in either the univariate or multivariate analysis.

To understand the key factors of early exit from work due to poor health, a prediction model was developed using the results from the Cox regression model. By stepwise elimination of the Cox regression model, literature review, and discussion with occupational and public health professions, finally, nine covariates were selected as the main features related to early exit from work due to poor health. These included age and sex among the basic characteristics; smoking and alcohol consumption among health behaviours; hypertension, diabetes, and abnormal BMI among disease statuses; and the perceived health and working life expectancy scores among the perception factors.

In detail, age, hypertension, abnormal BMI, and self-rated health status were selected by statistical significance in the Cox regression model and based on a previous study^8,9^. Sex was selected as an important covariate due to the fact that sex-differentiation can influence not only early exit from work decisions but also the later-life course^10^. Diabetes and both health behaviours (smoking and alcohol consumption) were added into the prediction model despite not showing statistical significance. In many countries, regular medical health examinations are conducted for working individuals to identify chronic disorders (hypertension, obesity, and diabetes) and poor health behaviours (smoking and alcohol). After discussion with occupational and public health professions, the final prediction model therefore included three non-significant medical examination-related factors: diabetes, smoking, and alcohol consumption level.

The HR (95% CI) of the working life expectancy score did not show obvious statistical significance; however, it indicated a tendency of a close link with early exit from work (HR, 0.94; 95% CI, 0.88–1.00 from the fully adjusted model). Furthermore, a previous study has indicated that working life expectancy scores can critically impact on later life and health^11^.

The grasping power index and occupational classification could not be parted in the prediction model, despite showing significant relationships with early exit from work. The grasping power index for general health is not yet established, although this score is frequently used to assess neurological motor function. Herein, we only could reveal the possibility of the grasping power index to be used as a later life-related factor. Moreover, green- and unskilled blue-collar workers showed significant vulnerability of early exit from work. However, their significant occupational classification was removed from the final prediction model due to the heterogeneity within each category such as in age and income.

HRs with exponentiated regression coefficients, obtained from the Cox regression model in Table 2, were used to build the risk score for the 8-year follow-up risk for non-voluntary early exit from work due to poor health. The early exit from work due to poor health risk score was constructed using beta estimates from the multivariate Cox regression analysis and the prevalence or mean of each predictive factor.

**Figure 1:**
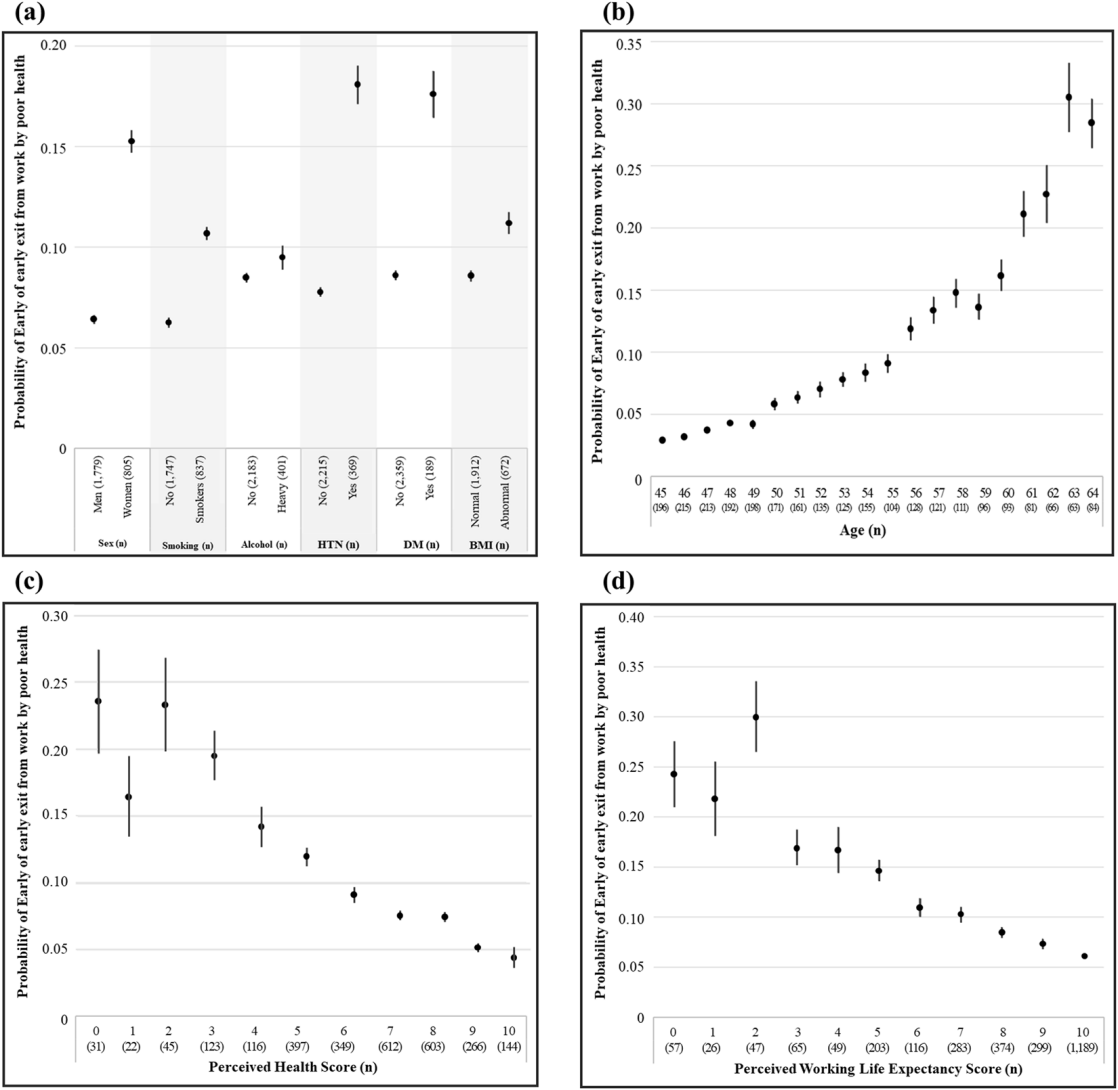
Mean probabilities of early exit from work by poor health according to (**a**) sex, health behaviours, and chronic disorders; (**b**) age; (**c**) perceived health score; and (**d**) perceived working life expectancy score. HTN, hypertension; DM, diabetes mellitus; BMI, body mass index.

Figure 1 demonstrates the mean and 95% CIs of the probability of early exit from work due to poor health estimated from the Cox regression model according to each predictive factor. Female workers, smokers, heavy alcohol consumption, and chronic disorders (hypertension, diabetes, and abnormal BMI) associated with high probabilities of early exit from work due to poor health. There were also the probabilities of early exit from work due to poor health according to increasing age, decreasing perceived health score, and the working life expectancy score.

### Performance of the Prediction Model for Early Exit from Work due to Poor Health

**Figure 2:**
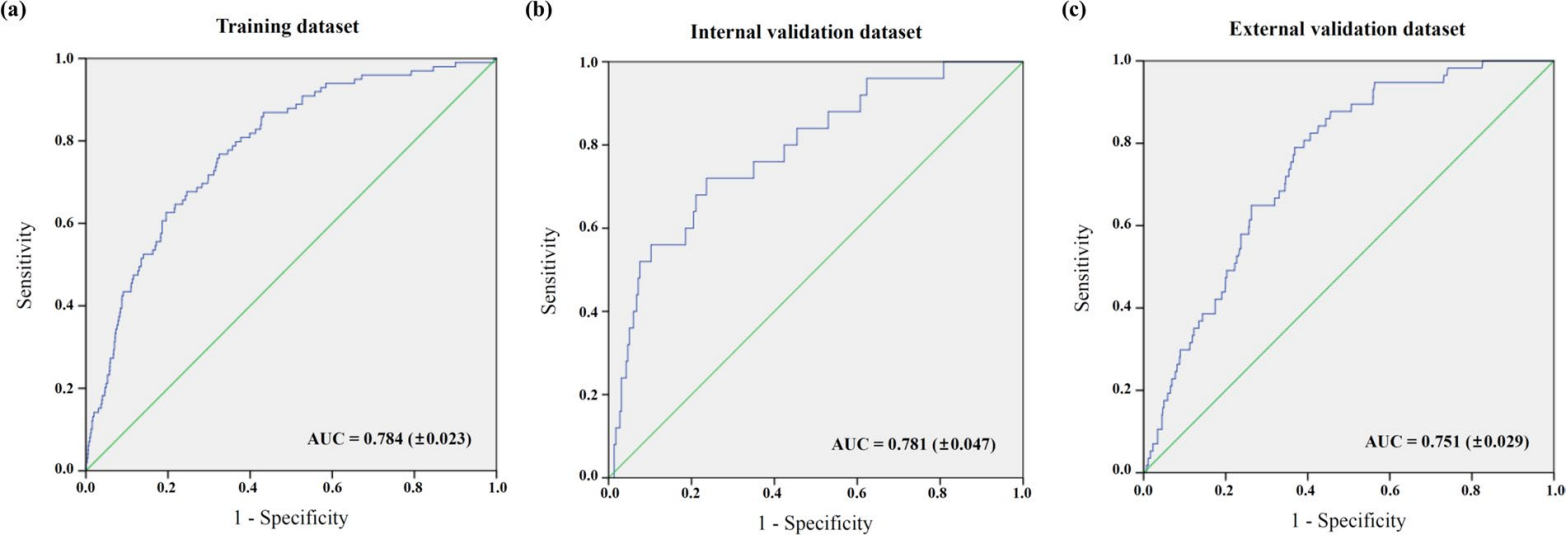
Areas under the curve (AUC) for early exit from work due to poor health. (**a**) Training, (**b**) internal validation, and (**c**) external validation datasets.

To evaluate the performance of the prediction model for early exit from work due to poor health, receiver operating characteristics (ROC) curve analysis with area under the curve (AUC) calculations were conducted in three different datasets, as summarized in Fig. 2. These included a (a) training dataset, (b) internal validation dataset from random split, and (c) external validation from the English Longitudinal Study of Ageing (ELSA). Our prediction model based on the Cox regression models showed fair AUCs of 0.784 (±0.023) in the training data set, 0.781 (±0.047) in the internal validation dataset, and 0.751 (±0.029) in the external validation dataset.

The predictive ability of the model was described by its sensitivity, specificity, positive predictive value, negative predictive value, and accuracy in each dataset, as summarized in Table 3. The internal validation dataset showed a sensitivity of 72.0%, specificity of 66.2%, positive predictive value of 9.3%, negative predictive value of 97.9%, and accuracy of 66.4%. The corresponding values in the external validation dataset were 94.7%, 63.0%, 6.4%, 99.7%, and 63.8%, respectively.

**Table 3:** Predictive ability of the model for early exit from work due to poor health.

	Internal validation	External validation
n	542	2,204
Sensitivity	72.0%	94.7%
Specificity	66.2%	63.0%
Positive predictive value	9.3%	6.4%
Negative predictive value	97.9%	99.7%
Positive likelihood ratio	2.12	2.56
Negative likelihood ratio	0.42	0.08
Accuracy	66.4%	63.8%

## Discussion

To our knowledge, this is the first attempt to create a prediction model of early exit from work due to poor health using various risk factors, including basic characteristics, health status, and perception factors. Specifically, we found that poor health status (hypertension and abnormal BMI) and low perceived health status were significantly associated with early exit from work due to poor health.

These results regarding the specific predictors of early exit from work due to poor health may help explain the findings of previous investigations. For example, chronic illness (hypertension or obesity) was reported as a risk factor of early exit from work due to poor health in a previous study using data from the Studies on Health and Retirement in Europe (SHARE)^12^. However, the current investigation further indicated that chronic disorders were significantly related with early exit from work due to poor health. Furthermore, other previous studies have reported limited associations between perceived health status and early exit from work^13^. The results from the current analysis suggest that perceived health status is significantly linked to early exit from work due to poor health.

Over 75% of ageing workers have been reported to choose to continue working even if they develop a significantly reduced work ability^14^. Unfortunately, the ageing process, which is often accompanied by disorders and inappropriate health behaviours, places burdens on the workers’ health that may cut their working life short^15,16^. Poor health conditions can lead to poor work performance, and both are important factors related to early exits from working life^17,18^. Indeed, to encourage a working life without poor health conditions, the risk for early exit from work due to poor health needs to be assessed.

In the present study, to identify the key obstacles to a sustainable working life, a statistical approach based on the current data and a literature review was used. As a result, this study identified nine factors as determinants of unwanted early exit from work due to poor health, including age, sex, health behaviours (smoking and alcohol consumption), chronic disorders (hypertension, diabetes, and abnormal BMI), and perception factors (health score and working life expectancy score).

Age is a fundamental component of researches in the elderly population, and plays a key role in early exit from work^9^. In the current analysis, the risk of early exit from work due to poor health showed a significant positive association with increasing age. The biological process of ageing is directly linked to worsened general health conditions, including work ability^19^. It is hard to stop the effects of ageing in humans; however, understanding the effects of age is a cornerstone in the evaluation of sustainable working life.

A much-debated question is whether health behaviours are related with early exit from work. Many studies, including the current one, were unable to demonstrate significant relationships, while others reported inverse relationships between smoking or alcohol consumption status and early exit from work^8,20^. However, one study focusing on early exit from work due to disability reported that smoking and alcohol were obvious risk factors^21^. In general, smoking and heavy consumption of alcohol are known risk factors for reduced workability and increased disability related to the workplace^22,23^. Furthermore, the workers’ smoking and alcohol consumption habits are some of the first questions during the regular medical check-ups for workers in Korea. Thus, this study considered cigarette smoking and alcohol consumption as predictive factors for early exit from work due to poor health.

According to previous researches and the present study, chronic disorders, including hypertension, diabetes, and abnormal BMI, can be strong factors associated with shortened working life^24–26^. In public health policies, these conditions are given priority, and, in clinical practice, evaluations of blood pressure, blood glucose, and anthropometry are fundamental in regular workers’ medical examinations.

The close linkage between perception and decision-making represents a well-established theory to explain human behaviour. Perception is a serial process of organizing and interpreting stimuli from the environment in order to give it meaning. Based on these interpretations and analyses of the situation, combined with a rational analysis, individuals will make a decision^27,28^. In occupational health, subjective perception is an important factor to understand workers’ health and behaviours. For example, workers who report higher perceived work stress and physical workload have been reported to be more likely to have neuromuscular disorders than those with lower scores^29,30^. Thus, we hypothesize that, in cases of early exit from work due to poor health cases, positive perceptions of health and sustainability of work life were unlikely to remain.

In the current study, decreased perceived health condition significantly associated with an increased risk for early exit from work due to poor health. Previous studies have also reported that perceived health status could be used as a predictor of unwanted early exit from work related to poor health^18,31,32^. Workers’ self-perceived health status deteriorates with age, and chronic diseases are common in older populations^33^. Moreover, participants in surveys related to old age are frequently asked about their subjective health status. Thus, using perceived health status is associated with several advantages for predicting the risk of early exit from work due to poor health.

There are currently limited published data on the relationship between perceived working life expectancy and early exit from work. However, the current study and a previous study by our group showed the possibility of using perceived working life expectancy as a predictor of early exit from work^11^. Using the exact working life expectancy score, similar to in the Korean Longitudinal Study of Ageing (KLoSA) and ELSA, a previous study reported that self-rated workability was closely related with unwanted exit from working life^34^. Hence, these two perception factors could potentially be applied to build a prediction model.

The current study suggested a novel prediction model for early exit from work due to poor health in older workers by considering nine core factors, including basic characteristics, health behaviours, chronic disorders, and perception factors. The performance of the prediction model was fair, and it was not attenuated even after external validation using data from the ELSA. More information regarding ageing workers at risk of early exit from work secondary to their health behaviours or chronic disorders may help increase the awareness of the importance of good health behaviours such as quitting smoking, reducing consumption of alcohol, and losing weight. It can thus be suggested that the prediction model from this study can be used to evaluate the risk of, or to identify vulnerable workers for, early exit from work due to poor health.

In many countries, occupational health is assessed through regular medical examinations (annually for labour workers and biennially in office workers) to prevent poor workers’ health. Nonetheless, many workers in this study could not remain in their workplace due to poor personal health conditions. This is also a main issue for lengthening working life, as poor health is, at least partially, preventable with appropriate knowledge and health management among elderly workers. Thus, occupational health professionals can also use our prediction model for early exit from work due to poor health to improve the workers’ health.

In general, it seems that, for understanding the process of shortened working life, determinants of early exit from work due to poor health should be considered. The current study has important implications on occupational health in that it revealed the possibility of prediction of unwanted early exit from work due to poor health. Predictability allows for the possibility of prevention. Taken together, our findings suggest a role of our prediction model as a preventive strategy of unwanted early exit from the workplace by poor health.

Nevertheless, despite these implications of our study, the results from the current analysis must be interpreted with caution because of the nature of the data. The survey data from the KLoSA were based on self-administered questionnaires. Surveys based on questionnaires are likely to have various errors such as specification, frame, nonresponse, measurement, and processing errors. Especially, self-rated perception-related questionnaires by retirees with poor health might also have bias. While the KLoSA applied computer-assisted interviews by trained researchers to reduce these errors, these limitations are not specific to research based on the KLoSA, and have been reported for surveys of the elderly population in various countries worldwide, including for the Health and Retirement Study (HRS), ELSA, and SHARE^35,36^. More subjective measurements and reports are needed to ensure the reliability and validity, as well as the practical applicability, of the KLoSA questionnaires. Furthermore, the small positive predictive value of our prediction model does not allow for the results from the current analysis to be immediately applied to workers. However, the prediction model of the current study showed moderate to good validity in terms of the sensitivity, specificity, and negative predictive value (Table 3), and good performance in terms of the AUCs (Fig. 2). Hence, we consider that this research may serve as a cornerstone for future studies about the prediction and prevention of unwanted early exit from workplace due to health problems. Finally, we researched exit from work focused on middle and older aged workers. However, the lack of jobs or forced retirement are same issues among younger adults who have poor health conditions too in many countries. More broad generation, research is also needed to understand early exit from work due to poor health process.

In conclusion, the present large-scale longitudinal study focusing on the older working population (including regular paid and self-employed workers) revealed several specific determinants of shortened work life because of poor health among workers. A prediction model from our investigation was proposed to evaluate the risk of early exit from work due to poor health. The performance of this prediction model was fair and was not attenuated even after external validation. Taken together, our results suggest that it is important to understand the determinants of early exit from work due to poor health, and estimations of the probabilities of unwanted early exit from work due to poor health condition should be considered a key factor in extending the working life in ageing workers.

## Methods

### Study Design and Data Collection

The current study used data from the first to fifth (2006–2014) waves of the Korean Longitudinal Study of Ageing (KLoSA), conducted by the Korea Labour Institute and the Korea Employment Institute Information Service. The KLoSA was conducted to obtain an overview of what it means to grow older and to help us understand what accounts for the variety of patterns that are seen. The KLoSA is a nationally representative panel survey of Korean citizens aged over 45 years. The panel survey, which has been conducted every 2 years since 2006, was built to provide a national resource for data on the changing age-related socioeconomic and health circumstances. Its multidisciplinary approach is focused on broad topics, including socioeconomics, health status, and relationships. The KLoSA was started with surveys and interviews of 10,254 randomly selected adults residing in one of 15 city-size administrative areas in the Republic of Korea in 2006. The KLoSA collects information about household and individual demographics; medical, physical, and psychosocial health; work and pensions; income and assets; housing; cognitive function; social participation and networks; expectations; and objective estimations, including physical and performance measures. The participants were interviewed using computer-assisted personal interviews, where the professional interviewers instructed the respondents to read the questions on a computer and input their answers directly.

**Figure 3:**
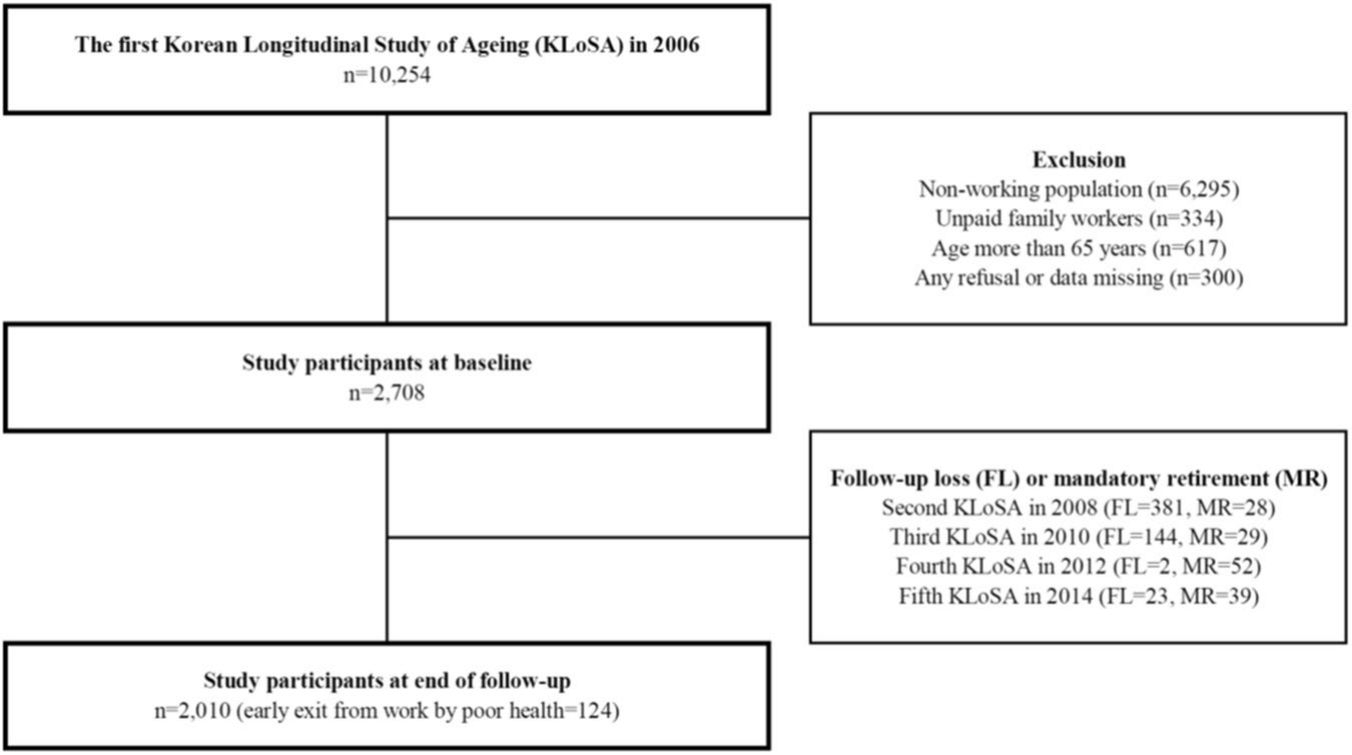
Schematic diagram of the study participants.

In this study, a longitudinal approach was adopted to develop an 8-year early exit from work prediction model; the baseline period used for this study was the first phase of the KLoSA (n = 10,254) conducted in 2006. In order to investigate the predictors of early exit from work, the non-working population (n = 6,295) and unpaid family workers (n = 334) were excluded from the study. Workers aged >65 years (n = 617) at baseline were also excluded according to the definition of early exit from work. Moreover, 300 participants who refused to participate or those with missing relevant covariates data were also excluded from the study. Finally, 2,708 workers were included in the current study at baseline (Fig. 3).

Each KLoSA participant was identified using a randomly selected number to ensure their anonymity. The interviewers provided information about the research objectives and potential risks and benefits to all survey respondents, who provided informed consent before they answered any questions. All respondents also agreed to participate in further scientific research. The present study was approved by the Institutional Review Board of Yonsei University Graduate School of Public Health, Korea (No. 2-1040939-AB-N-01-2016-167). This study followed the STROBE (Strengthening The Reporting of OBservational Studies in Epidemiology) reporting guidelines for observational cohort studies.

### Early Exit from Work

Early exit from work can be defined as retirement before normal pension age. In Korea, the beginning ages of normal pension age are separated according to the year of birth (~1952, 1952–1962, 1963–1964, and 1965~). Most people born after 1965 will retire at 65 years. Workers who retire before this age are defined as early retirement workers. Early exit from work may also be defined as retirees who retire despite the ability and will to work due to various reasons. According to the Organization for Economic Cooperation and Development (OECD) reports in 2017 (http://www.oecd-ilibrary.org/finance-and-investment/oecd-pensions-at-a-glance_19991363;jsessionid=1ckwr6b4gmbr5.x-oecd-live-02), the average effective age of labour market exit was 65.1 years for men and 63.6 years for women across OECD countries. In Korea, the average effective age of labour market exit was the highest among the OECD countries (72.0 years for men and 72.2 years for women). Considering the various definitions of early exit from work and social circumstances in Korea, in this study, we defined early exit from work using a less strict standard, as retirement before 65 years.

In order to identify early exit from work due to poor health, newly recognized retirees during the follow-up periods were asked regarding the reason for retirement in each wave. The possible answers were categorized as follows: (1) sufficient income, (2) sufficient income of their spouse, (3) being weary of work, (4) wanting to have more leisure time, (5) wanting to spend time volunteering or on a hobby, (6) poor personal health status, (7) poor health of their spouse, (8) poor health of family members, (9) housework or childcare, (10) unable to find other work, (11) regular retirement, and (12) any other reasons. Participants who answered with 11 and 12 were excluded from the study, as these cases were considered mandatory retirement.

The retired participants who answered ‘(6) due to poor personal health status’ were defined as ‘early exit from work due to poor health.’

### Socioeconomic Variables

The current study used age, sex, marital status, education, and household income as socioeconomic variables. Marital status was divided into two categories (married vs. divorced, separated, or never). Educational level was divided into the following three categories: less than middle school graduation, high school graduation, and above. Household income level was categorized as follows: <$20,000, <$30,000, <$40,000, and ≥$40,000.

### Occupational Characteristics

Occupational characteristics included the type of work, occupational classification, size of enterprise, and time of work. The occupational classifications were regrouped into six out of the 10 major categories (suggested by the International Standard Classifications of Occupations, as per the social and cultural circumstances of the Republic of Korea as well as the skill and duty levels reported in a previous study^37^), as follows: higher-skilled white-collar workers (legislators, senior officials, managers, and professionals), lower-skilled white-collar workers (technicians and associated professionals), pink-collar workers (clerk, sales, and customer service workers), green-collar workers (agriculture, fishery, and forestry), skilled blue-collar workers (craft, plant and machine operators, and assemblers), and unskilled blue-collar workers (elementary workers). The size of the enterprise was divided into two categories considering the type of work (regular paid workers and self-employed), as follows: self-employed one-man companies and companies with <30 workers among paid workers were grouped into same category, while all other enterprises were grouped together.

### Health Status

Smoking, alcohol consumption, and exercise level were considered as health behaviour-related covariates. Smoking history was categorized as never/past smokers or current smokers. Heavy alcohol consumption was defined as alcohol consumption of at least 15 standard drinks per week in men and at least 8 standard drinks per week in women. Regular exercise was defined as exercising more than once a week, as determined using the questionnaire.

The KLoSA included information about the participants’ medical history. Diagnoses of hypertension, diabetes, depression, and measured abnormal BMI (≥25 kg/m^2^, obesity; <18.5 kg/m^2^, underweight) were included as relevant disease histories of the participants, as chronic diseases can lead to work disability in the older population^33,38^.

The grasping power index reflects age-related health conditions, similar to the gait speed and balancing on one-foot tests. For each hand, the mean of three trials of grip strength was calculated while considering the participants’ conditions for gripping and hand dominance^39^.

### Perception Factors

The KLoSA included questionnaires about the self-rated expectations or satisfactions of the participants. The current study used three satisfaction factors (health, economic, and quality of life) and one expectation factor (working life expectancy). Self-rated satisfaction levels are considered key aspects related to unwanted early exit from work among elderly workers^40,41^. The participants were asked about their health/economic/quality of life satisfaction as follows: “How satisfied are you with your health/economic/quality of life status?” Working life expectancy has also been reported as an important factor related to the ageing population^11^. Questions about the working life expectancy were asked to the participants according to their age group, using the following statements: “I can keep working in this job until 55 years old” to those <50 years of age, “I can keep working in this job until 60 years old” to those aged between 50–54 years, and “I can keep working in this job for 5 more years” to those aged >55 years. The possible answers to these statements were provided using a visual analogue scale (0–10 points). A score of 0 signified “never” or “it will never happen to me”, while a score of 10 signified “always” or “it will definitely happen to me.”

### Statistical Analysis

The frequency of unwanted early exit from work due to poor health was calculated for each data category, and the chi-squared test or t-test was used to evaluate the association between each variable and early exit from work. The survival time was defined as the interval between the survey date of the first wave and the date of early exit from work due to poor health. Hazard ratios (HRs) and 95% confidence intervals (CIs) were calculated by Cox regression models to evaluate the risks for early exit from work due to poor health.

A Cox regression model was also used to construct a prediction model for the 8-year probability of early exit from work due to poor health as a means to identify vulnerable workers and to provide information related to unexpected early exit from work. A functional formula for probabilities of early exit from work due to poor health according can see in Supplementary Table 1.

For the feature selection, backward stepwise elimination was conducted along with a literature review and expert discussion.

For internal validation, the dataset was divided randomly into training (80%, n = 2,166) and test sets (20%, n = 542), with conservation of the prevalence of early exit from work due to poor health. The KLoSA was developed as part of a research network with the ‘Health and Retirement Study (HRS)’ in the US, ‘Studies on Health and Retirement in Europe (SHARE)’ in the EU, and ‘English Longitudinal Study of Ageing (ELSA)’ in the UK. These studies were designed to share key areas of research, reflect cultural circumstances, and use understandable measurements, and are international comparable studies to the KLoSA^42^. Thus, for external validation, data from the ELSA were used. The ELSA is a panel study of a representative cohort of the elderly population living in England^43^. It was designed as a sister study to the HRS and contains multidisciplinary data, with a similar structure to the KLoSA. The panel was initially held in 2002, and the participants (>10,000) have been followed-up every 2 years. We used data from 2006 to 2014 of the ELSA to match the follow-up periods of the current analysis data from the KLoSA. A total of 2,204 participants were selected under the same conditions as for current analysis dataset from the KLoSA.

Area under the curve (AUC) and receiver operating characteristic (ROC) curve analyses were conducted to evaluate the performance of the prediction model for early exit from work due to poor health. All statistical analyses were performed with SAS (version 9.4; SAS Institute, Cary, NC, USA). Two-tailed p values of <0.05 were considered to indicate statistical significance.

## Electronic supplementary material

Supplementary table 1
