# Supplementary material for: Predictors and estimation of risk for early exit from working life by poor health among middle and older aged workers in Korea
Source: Sci Rep. 2018 Mar 26;8:5180. doi: 10.1038/s41598-018-23523-y (PMC5979952; doi:10.1038/s41598-018-23523-y)
Supplement: Supplementary file 1 — Supplementary table 1 [file 41598_2018_23523_MOESM1_ESM.docx]

**Title:** Predictors and estimation of risk for early exit from working life by poor health among middle and older aged workers in Korea.

**Authors**

Wanhyung Lee, M.D., Ph.D.^1,2,3^, Jin-Ha Yoon, M.D., Ph.D.^1,2,3,4^, Jung-Wan Koo, M.D., Ph.D.^5^, Sei-Jin Chang, Ph.D.^6^, Jaehoon Roh, M.D., Ph.D.^1,2,3,4^ and *Jong-Uk Won, M.D., Ph.D.^1,2,3,4^

**Affiliations**

^1^The Institute for Occupational Health, College of Medicine, Yonsei University, Seoul, Korea

^2^Graduate School of Public Health, College of Medicine, Yonsei University, Seoul, Korea

^3^Incheon Worker’s Health Center, Incheon, Korea

^4^Department of Preventive Medicine, College of Medicine, Yonsei University, Seoul, Korea

^5^Department of Occupational and Environmental Medicine, Seoul St. Mary`s Hospital, College of Medicine, The Catholic University of Korea, Seoul, Korea

^6^Department of Preventive Medicine, Institute Occupational and Environmental Medicine, Yonsei University Wonju College of Medicine, Wonju, Korea.

***Correspondence to this address:**

Jong-Uk Won M.D., Ph.D.

The Institute for Occupational Health, Department of Preventive Medicine,

College of Medicine, Yonsei University,

50, Yonsei-ro, Seodaemun-gu, Seoul, Korea, [03722]

Tel: +82-2-361-5375; Fax: +82-2-392-8622

E-mail: ys.[juwon@gmail.com](mailto:juwon@gmail.com)

**Supplementary**

| Supplementary table 1. The early exit from work due to poor health risk score |
| --- |
| *Probability(x) = (0.12465)*(AGE-52.5489381) +(1.02939)*(SEX-0.3236) +(0.00648)*(SMOKING-0.3163) +(0.13462)*(ALCOHOL-0.1565) +(0.37181)*(DM-0.0711) +(0.38628)*(HTN-0.1477) +(0.09464)*(BMI-0.2715) +(-0.09659)*(S_HEALTH-6.6629732) +(-0.08266)*(S_WORKING-8.0452447).* |
| AGE is a continuous variable, SEX has two categories (men or women); SMOKING has two categories (never or past/current); ALCOHOL has two categories (never or social/heavy); DM and HTN are diagnosed or treated diabetes mellitus or hypertension, respectively; BMI is an abnormal BMI (≥25 kg/m^2^: obesity and <18.5 kg/m^2^: underweight); S_HEALTH is the perceived health score (0–10 points with 1-point intervals); and S_WORKING is the perceived working life expectancy score (0–10 points with 1-point intervals). |
